# Multicentre evaluation of the IL Densiscan

**DOI:** 10.1155/S1463924686000056

**Published:** 1986

**Authors:** P. A. Bonini, E. Callioni, F. Ceriotti, M. Murone, F. Aguzzi, A. Milanesi, E. Ferrario

**Affiliations:** 1 Ist Scientifico Ospedale S. Raffaele Via Olgettina 60 Milan 20132 Italy; 2 Ospedale di Broni Stradella Pavia Italy; 3 Instrumentation Laboratory S.p.A. Milano Italy


Journal of Automatic Chemistry/*Journal of Clinical Laboratory Automation*, Volume 8, Number 1 (January–March 1986), pages 18–22

### Multicentre evaluation of the IL Densiscan

**P. A. Bonini, E. Callioni, F. Ceriotti, M. Murone,**
*Ist Scientifico Ospedale S. Raffaele, Via Olgettina 60, 20132 Milan, Italy*
**F. Aguzzi, A. Milanesi**
*Ospedale di Broni e Stradella, Pavia, Italy*
**and E. Ferrario**
*Instrumentation Laboratory S.p.A., Milano, Italy*

### Introduction

The electrophoretic separation of human proteins (for example serum proteins and lipoproteins) is a very popular test in clinical chemistry; separation is usually followed by a densitometric reading. In this paper the characteristics and performance of a new automatic densitometer are described (the IL Densiscan, Instrumentation Laboratory SpA, Italy), together with the results of an evaluation. It was not possible to follow any international guideline or recommendation – there is no accepted international standard for the description and evaluation of densitometers. Therefore the experiments were developed in comparison with other kinds of instrument; some general statements in the literature were also followed [1 and 2].

Precision and resolution – which define the accuracy of minima point detection – were specifically investigated. Any interference from the quality of the electrophoretic migration on the performance of the instrument was ruled out.

### Materials and methods

### *Instrument design*

*Densitometer hardware:* the IL Densiscan is a fully automatic instrument designed for the analysis of a single slide at a time. The instrument is programmable for scanning a variety of electrophoretic separations in micro, semimicro and macro size on a number of supports, which include cellulose acetate, agarose, poliacrylamide. Both transparent and partially transparent supports can be analysed.

The optical system of the instrument consists of an halogen lamp for scanning in transmission only and two filters, 525 and 620 nm. No fluorescence is possible. The analysis time is 20s per sample (including print-out of results). A photodetector transforms the emerging light beam into an electrical signal, which is then amplified, digitized and converted in accordance with a fast Fourier transform algorithm. After the results have been calculated, the signal is reconverted to a digital form for printing. An alphanumeric printer and a simple display system provide for a comprehensive dialogue between the operator and the instrument. A schematic diagram of the instrument is shown in figure 1.

*Densitometer software:* a general program is available for any kind of electrophoretic pattern; a graph is supplied with minima points identified (no identification is given when an inflexion in the curve is found). The percentages of the fractions are indicated underneath each graph. There is also a dedicated program for serum protein pherograms. The scanning movement covers the gamma area to albumin and all data are stored. Serum protein fraction identification is carried out as follows: the first fraction, if greater than 20%, is identified as albumin (pre-albumin, if any, is included in the albumin fraction). The following two areas are considered as $\alpha_1$ and $\alpha_2$. Then two areas are identified as $\beta_1$ and $\beta_2$, if necessary allocating a value ø to $\beta_2$, if $\beta_1$ and $\beta_2$ are not separated. The remaining area is indicated as $\gamma$; any monoclonal component, migrating in gamma region, is included in the gamma fraction. No further identification is made if more than six fractions are read—a list of unidentified fractions with their percentages is given. A procedure is available for manual correction of minima points without the need to rescan the sample.

The software also includes a quality-control program to check instrument precision avoiding any interference connected with electrophoretic migration. A single pherogram is automatically scanned 32 times; the mean, the

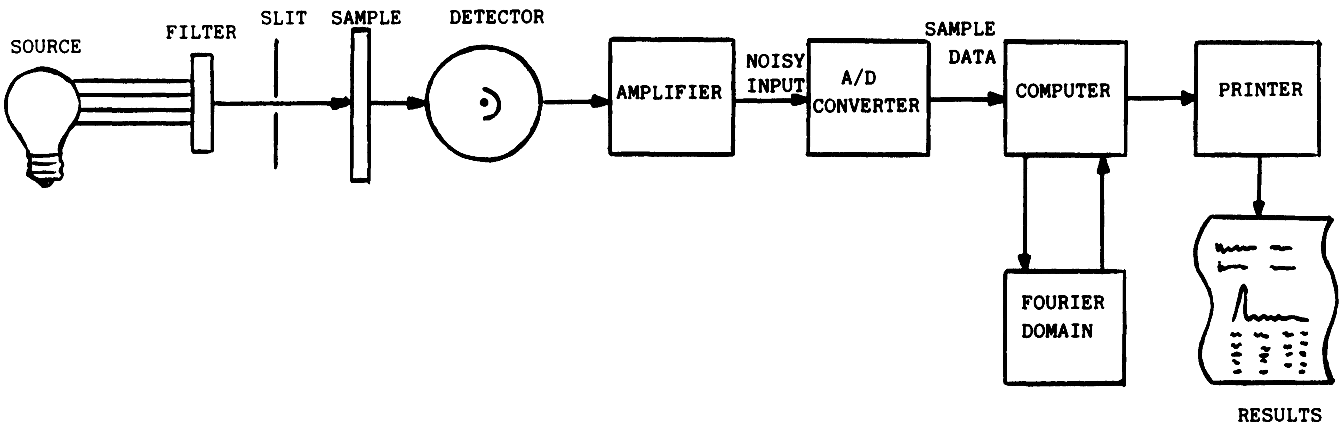

*Figure 1. Schematic description of the IL Densiscan.*

standard deviation and the coefficient of variation are given for each fraction. This program can only be applied to serum protein pherograms.

*Methematical treatment of data*

Minima detection is a crucial feature of densitometers because of the high noise interference of the signal due to the characteristics of the different electrophoretic supports. To avoid this, the analogue signal is usually filtered at the output of the amplifier. In most densitometers the filtering function is fixed and does not take into account the different backgrounds which occur with different supports. This results in a limited capability of minima detection with manual correction often required. In the IL Densiscan the digital filtering function is optimized by adopting a fast Fourier transform algorithm. In this way filtration is modified by the background produced by the support, resulting in a better identification of minima points (figure 2).

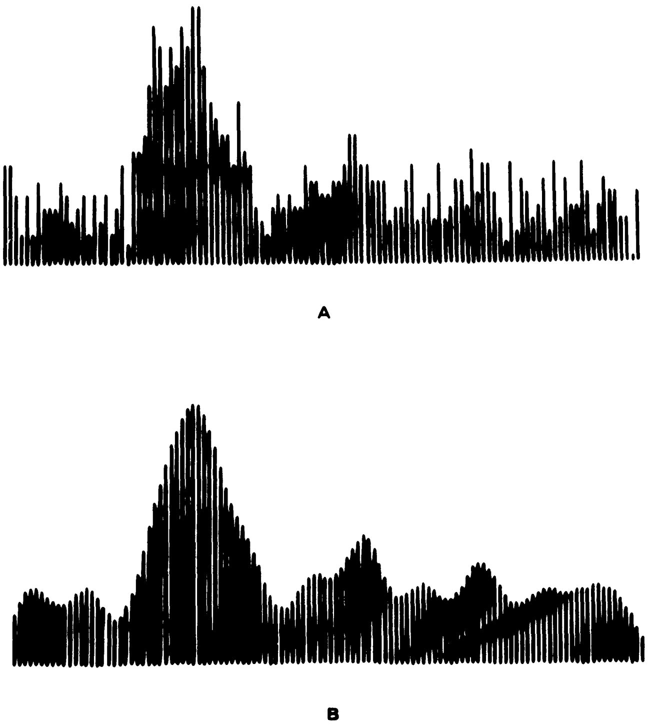

*Figure 2 (a). Example of a pherogram scan before filtering using using the fast Fourier transform. (b) Example of a pherogram after filtering using the fast Fourier transform.*

### Evaluation

*Pre-marketing evaluation*

Three Italian clinical laboratories were supplied with three prototypes of the instrument and were asked (after a short training period) to use them for a month with their own equipment. No strict evaluation protocol was followed; operators were asked to repeat their routine work with the prototype and to report on performance, ease of operation, drawbacks and problems. A generally positive conclusion was drawn by all participants.

*Main evaluation*

Two laboratories (Ospedale S. Raffaele, Milano, and Ospedale Civile Stradella, Pavia) were given one instrument each from the production line. In the first laboratory, instrument precision, accuracy of the slide-positioning system and the capability of minima detection were evaluated. The second laboratory checked the sensitivity and the criteria of serum protein fraction identification.

Electrophoretic separations were obtained on routine samples for serum proteins, lipoproteins and haemoglobins following the procedures described in table 1.

*Table 1. Separation procedure.*

| | |
|---|---|
| First laboratory (Ospedale S. Raffaele, Milano) | |
| *Serum protein:* | cellulose acetate Titan III-Helena; buffer Tris barbital pH 8.9–9.0 50 mmol/l; red Ponceau S; migration 200 V for 25 min. |
| *Lipoproteins:* | agarose Corning; buffer barbital pH 8.6 50 mmol/l; oil red; migration 90 V for 35 min. |
| *Haemoglobins:* | cellulose acetate Titan III-Helena; buffer Tris EDTA pH 8.2–8.6 25 mmol/l; red Ponceau S; migration 350 V for 23 min. |
| Second laboratory (Ospedale Civile Stradella, Pavia) | |
| *Serum protein:* | cellulose acetate Proteo-Elvi; buffer barbital 50 mmol/l; Amido Schwarz; migration length 4,5 cm |

### Experimental and results

*First laboratory*

Electrophoretic separations were obtained on 11 specimen for serum proteins (three containing monoclonal components, two with increased gamma fraction and six normals), 10 for lipoproteins and five for haemoglobins. Each pherogram was scanned 32 times a day for five consecutive days on the IL Densiscan and on a reference instrument.

The overall mean value for each fraction obtained on both instruments is reported in table 2 (serum proteins), table 3 (serum lipoproteins) and table 4 (haemoglobins): there is no evidence of discrepancy between the instruments.

To evaluate the imprecision of each instrument, CVs within series, between series and overall were calculated for each electrophoretic fraction in each sample.

To obtain a synthetic expression of the imprecision on different kinds of samples, the mean CV was calculated with the following formula:

$$\sqrt{\sum_{i=1}^{n} CV^2/n}$$

*Table 2. Overall mean value for each fraction obtained using the IL Densiscan (IL) and the reference instrument (R). Mean CV within day (W), between days (b) and overall (o) for serum proteins. Student t-test as follows: ns (not significant), *** $p < 0.01$, * $p < 0.02$, * $p < 0.05$. § The mean CV was calculated by the formula:*

$$\sqrt{\sum_{i=1}^{n} CV^2/n}$$

| | Albumin | | $\alpha_1$ | | $\alpha_2$ | | β | | Gamma | |
|---|---|---|---|---|---|---|---|---|---|---|
| | IL | R | IL | R | IL | R | IL | R | IL | R |
| Mean (11 samples) % | 49·89 | 50·00 | 3·45 | 3·58 | 9·40 | 9·30 | 12·36 | 12·40 | 24·86 | 24·80 |
| § $CV_w$ | 0·54** | 1·29 | 1·54** | 4·56 | 2·62 ns | 6·67 | 2·27*** | 3·78 | 1·79* | 3·09 |
| § $CV_b$ | 1·09** | 4·56 | 3·06** | 6·34 | 1·92** | 5·25 | 2·12 ns | 9·86 | 2·76 ns | 11·01 |
| § $CV_0$ | 1·37*** | 4·73 | 3·34*** | 7·73 | 3·23 ns | 8·40 | 3·07 ns | 10·54 | 3·18 ns | 12·84 |

*Table 3. As table 2 – for lipoproteins.*

| | Alpha | | preBeta | | Beta | |
|---|---|---|---|---|---|---|
| | IL | R | IL | R | IL | R |
| Mean (10 samples) % | 29·80 | 27·94 | 24·98 | 26·50 | 45·30 | 45·50 |
| § $CV_w$ | 1·22*** | 2·80 | 2·91 ns | 5·18 | 1·55 ns | 2·25 |
| § $CV_b$ | 3·48 ns | 3·59 | 4·03 ns | 6·97 | 5·30 ns | 3·52 |
| § $CV_0$ | 3·68 ns | 4·55 | 4·96 ns | 8·67 | 5·44 ns | 4·16 |

*Table 4. As table 2 – for haemoglobin.*

| | $A_1$ | | $A_2$ | |
|---|---|---|---|---|
| | IL | R | IL | R |
| Mean (5 samples) % | 95·29 | 94·93 | 4·71 | 5·07 |
| $CV_w$ | 0·32* | 0·2 | 5·40 ns | 3·57 |
| $CV_b$ | 0·60 ns | 1·93 | 11·77 ns | 28·05 |
| $CV_0$ | 0·68 ns | 1·94 | 12·81 ns | 28·22 |

A comparison between the CVs obtained with the two instruments was performed using the Student t-test for paired data (see tables 2–4). For serum proteins (table 2), the CVs for albumin and $\alpha_1$ measurements on the IL Densiscan differ significantly from those for the reference instrument. For $\alpha_2$, β and γ the lack of statistical significance was caused by incorrect results for samples 5 and 8 from the reference instrument.

No statistically significant differences were found for lipoproteins and haemoglobins.

*Table 5. CV within day (w), between days (b) and overall (o) for serum proteins using the IL Densiscan (IL) and the reference instrument (R), repositioning the slide each time.*

| | | Albumin | | α1 | | α2 | | β | | γ | |
|---|---|---|---|---|---|---|---|---|---|---|---|
| Samples | CV | IL | R | IL | R | IL | R | IL | R | IL | R |
| 1 | w | 1·61 | 2·73 | 6·23 | 3·57 | 2·32 | 2·16 | 4·54 | 6·69 | 2·41 | 4·00 |
| | b | 0·36 | 1·11 | 0·62 | 0·22 | 0·16 | 0·543 | 1·23 | 1·26 | 0·61 | 1·93 |
| | o | 1·65 | 2·94 | 6·26 | 3·57 | 2·33 | 2·16 | 4·63 | 6·79 | 2·48 | 4·41 |
| 2 | w | 3·09 | 3·95 | 7·43 | 10·94 | 3·91 | 5·99 | 3·40 | 25·96 | 15·02 | 21·56 |
| | b | 1·10 | 1·78 | 4·00 | 2·59 | 2·48 | 1·72 | 1·01 | 4·60 | 9·03 | 12·14 |
| | o | 3·28 | 4·32 | 8·29 | 11·23 | 4·59 | 6·18 | 3·49 | 26·34 | 17·50 | 24·67 |

Repetitive readings were normally made without repositioning pherograms, the influence on the precision of refitting the same slide into the instrument was tested using two serum protein pherograms. They were scanned 32 times on each of three consecutive days, repositioning them for each scan both on the IL Densiscan and on the reference instrument. The results are shown in table 5. No statistical evaluation was performed because of the limited numbers in these experiments. The capability of minima detection was tested by scanning abnormal pherograms with poor resolution between fractions (tables 6–8).

Each pherogram was scanned 32 times and the performance of the instrument was evaluated by recording how many times the operator had to correct the results; comparing the findings with those on the reference instrument.

*Table 6. Percentage of correct automatic minima point detection for serum proteins.*

| Samples | IL(%) | R(%) |
|---|---|---|
| 1 | 100 | 100 |
| 2 | 100 | 100 |
| 3 | 100 | 100 |
| 4 | 100 | 83·7 |
| 5 | 100 | 61·2 |
| 6 | 100 | 98 |
| 7 | 100 | 98 |
| 8 | 100 | 92·5 |
| 9 | 100 | 96 |
| 10 | 93 | 90 |
| 11 | 95 | 66 |

*Table 7. Percentage of correct automatic minima point detection for lipoproteins.*

| Samples | IL(%) | R(%) |
|---|---|---|
| 1 | 100 | 100 |
| 2 | 100 | 100 |
| 3 | 100 | 100 |
| 4 | 100 | 100 |
| 5 | 100 | 100 |
| 6 | 100 | 100 |
| 7 | 100 | 99 |
| 8 | 76 | 74 |
| 10 | 100 | 98 |
| 11 | 100 | 93 |

*Table 8. Percentage of correct automatic minima point detection for haemoglobin.*

| Samples | IL(%) | R(%) |
|---|---|---|
| 1 | 100 | 100 |
| 2 | 100 | 100 |
| 3 | 100 | 100 |
| 4 | 100 | 100 |
| 5 | 100 | 100 |

*Second laboratory*

To investigated instrument sensitivity, 1:2 1:4 and 1:8 dilutions of a normal serum were prepared and the pherograms obtained with each solution were scanned. The results are shown in table 9. Additionally, the limits of the dedicated program for serum proteins was studied in 30 pherograms characterized by various abnormalities: eterozygosity, splitting of $\alpha_2$ zone, and presence of monoclonal components. Unless there is a sufficient change of slope between an abnormal and normal fraction, no identification of the abnormal fraction is possible. Usually, when more than six fractions are present they are not automatically identified, instead they are listed together with their percentage value.

### Discussion

The evaluation concentrated on instrument precision, capability of minima detection, sensitivity and validity of the program for serum proteins. The results shown in tables 2–4 indicate a high instrument precision in comparison with the reference instrument, both for serum proteins, lipoproteins and haemoglobins.

It appears that the high precision of the instrument is due to the data-processing method (fast Fourier transform), to the capability of minima detection and also to the system of slide positioning. Even refitting slides for each scan does not reduce precision (see table 5). Tables 6, 7 and 8 show that automatic minima detection, in comparison with the reference instrument, is excellent. Table 9 demonstrates that at low protein concentration the most heterogenous zones of the pherograms ($\beta$ and $\gamma$) give low values. This is probably due to the quantity of linked dye being lower than the instrument sensitivity limit, so that, to achieve success, electrophoresis of specimens with low protein concentrations, for example urine, require a preliminary concentration to at least 4 g/dl total protein.

Automatic fraction identification by a dedicated software system is easy and saves time, but does not do away with

*Table 9. Effect of sample dilution on serum protein electrophoresis.*

| Dilution | Total protein g/dl | Alb. % | Alfa 1 % | Alfa 2 % | Beta % | Gamma % |
|---|---|---|---|---|---|---|
| | 7·6 | 47·63 | 5·0 | 11·58 | 18·55 | 16·84 |
| 1:2 | 3·8 | 51·97 | 5·13 | 10·79 | 15·79 | 16·05 |
| 1:4 | 1·9 | 53·82 | 6·71 | 11·45 | 15·0 | 13·55 |
| 1:8 | ·95 | 59·61 | 7·5 | 13·45 | 11·45 | 7·76 |

the necessity for the operator to inspect each pherogram [3]. The software does not identify abnormal zones (eterozigosity and monoclonal components for example). However, the instrument can be recommended as easy to operate and appropriate for a medium-size laboratory's work-load.

### References

1. Alpert, N., *Laboratory World*, **28**, 20 (1977).
2. Alpert, N., *Laboratory World*, **28**, 20 (1977).
3. Aguzzi, F., Jayakar, S. D., Merlini, G. and Petrini, C., *Clinical Chemistry*, **27** (1981), 1944.

### EASTERN ANALYTICAL SYMPOSIUM TO CELEBRATE SILVER JUBILEE

As reflected by a substantial rise in attendance over the last few years, the Eastern Analytical Symposium has established itself as a significant national and international event in the scientific community.

1986 will mark the 25th annual EAS, which has been christened the Silver Jubilee. To mark this occasion, the EAS Governing Board has authorized the first-ever five-day EAS. A total of 50 technical sessions are planned for the five days of the meeting, as well as the traditional EAS poster sessions which will be held each day, giving a substantial increase in the number of papers to be presented at the Silver Jubilee.

In conjunction with the celebration of the Silver Jubilee, the EAS will present the First Eastern Analytical Symposium Award for Outstanding Contributions to the Fields of Analytical Chemistry.

The Silver Jubilee will be the first EAS to take place at the New York Hilton Hotel; the facilities at the Hilton will permit the expansion of the exposition to accommodate a greater variety of exhibits than has heretofore been possible, while permitting the exposition to take place in a location exceptionally convenient to all meeting rooms.

With the move to the Hilton, the EAS has been able to finalize its meeting dates for the next five years—

1986: October 6–October 10
1987: September 14–September 18
1988: September 26–September 30
1989: September 25–September 29
1990: September 24–September 28

*Call for papers*

A limited number of oral and poster presentations on new developments in analytical chemistry will be accepted for the Symposium. These contributed presentations will be grouped into several sessions to complement the invited technical sessions. Prospective authors should submit a 50– to 100–word abstract on the proposed presentation before 15 February 1986, indicating preference of oral or poster format, to Concetta M. Paralusz, EAS Program Chairman, Permacel/Avery International, P.O. Box 671, New Brunswick, New Jersey 08903; tel.: 201 524 5633. Care should be excercised in considering the title and authors of the proposed presentation; if the presentation is accepted, both title and authors will be considered final and not subject to change. Authors of accepted presentations will receive forms for submission of a 200- to 300-word abstract which will appear in the final program.

*Details (exhibiting and attending) from Dr S. David Klein, EAS Publicity, 642 Cranbury Cross Road, North Brunswick, New Jersey 08902, USA. Tel.: 201 846 1582.*

